# Raman spectroscopic analysis of carbonates on a CY-type carbonaceous chondrite Yamato 980115: unexpectedly abundant terrestrial weathering products

**DOI:** 10.1007/s44211-025-00731-x

**Published:** 2025-02-24

**Authors:** Shu-hei Urashima, Hayato Tsuchiya, Naoya Imae, Akira Yamaguchi, Hiroharu Yui

**Affiliations:** 1 Water Frontier Research Center, Research Institute for Science & Technology, Tokyo University of Science, 1-3 Kagurazaka, Shinjuku, Tokyo 162 8601 Japan; 2 Department of Chemistry, Faculty of Science, Tokyo University of Science, 1-3 Kagurazaka, Shinjuku, Tokyo 162-8601 Japan; 3 National Institute of Polar Research (NIPR), 10-3 Midori-cho, Tachikawa, Tokyo 190-8518 Japan; 4 The Graduate University for Advanced Studies (SOKENDAI), 10-3 Midori-cho, Tachikawa, 190-8518 Japan; 5 Nuclear Science and Engineering Center, Japan Atomic Energy Agency (JAEA), Tokai, Ibaraki 319-1195 Japan

**Keywords:** Antarctic meteorites, Terrestrial weathering, Carbonates, Raman microscopy

## Abstract

This study presents Raman spectra of carbonates on a carbonaceous chondrite, Yamato (Y) 980115. Y 980115 is an Antarctic meteorite and is originally classified as CI1-type. Since CI1-type meteorites are expected to record information of aqueous alteration of carbonaceous matters in the primitive solar photosphere or planetesimals, extensive studies have been performed. Among a variety of minerals on meteorites, carbonates are one of environment indicators because its cation composition depends on thermochemical properties of the aqueous solution when they were precipitated. Although the minerals on meteorites are usually identified by elemental composition analysis techniques such as backscattered electron imaging, these techniques have a drawback that they lack information of crystal structures so that it is difficult to distinguish some minerals such as ordered or disordered dolomite (CaMg(CO_3_)_2_) and a mixture of calcite (CaCO_3_) and magnesite (MgCO_3_). The present study employed Raman spectroscopy as a complementary technique to the elemental composition analysis. As a result, surprisingly, while the Mg-bearing carbonates on Y 980115 were expected mainly to be dolomite, the Raman spectra revealed that they are indeed nesquehonite (Mg(HCO_3_)(OH)·2H_2_O), which is a typical terrestrial weathering product for Antarctic meteorites. Among 63 carbonate grains found on Y 980115, 60 and 3 grains were assigned to nesquehonite and calcite, respectively, and no dolomite was found. This result indicates that there are unexpectedly abundant terrestrial weathering products for carbonates found on Y 980115.

**Graphical abstract:**

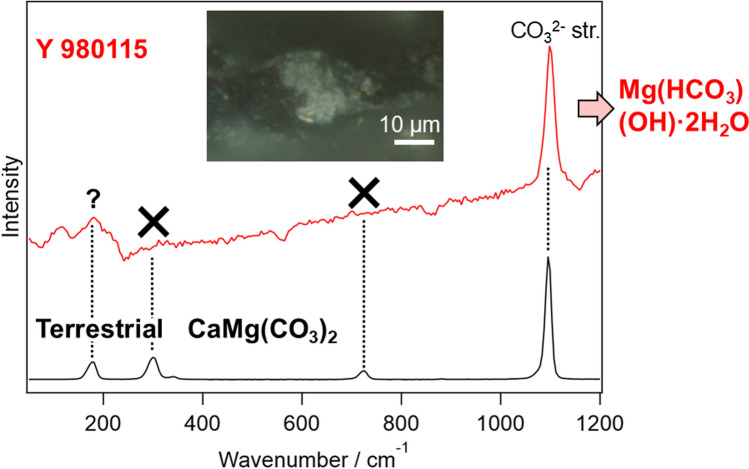

## Introduction

Carbonaceous chondrites, which are primitive meteorites, have been extensively studied to unveil chemical evolution of carbon and water in protosolar systems [1]. While they are classified as, e.g., CI1, CM2, and CV3 types based on their textures, chemical and oxygen isotopic compositions, and their alteration history, the CI1 chondrites have gotten great attention because their parent bodies are considered to preserve elemental compositions in the primitive solar photosphere and profoundly experience aqueous alteration on planetesimals. Among ~ 76,000 meteorites found so far, only 9 meteorites, i.e., Alais, Ivuna, Orgueil, Revelstoke, Tonk, Y-86029, Y-86737, Y 980115, and Y 980134, are officially classified as CI1 [2]. The rarity of the CI1 type chondrites was one of the motivations for the JAXA Hayabusa 2 project to collect regolith from the asteroid 162173 Ryugu, which had been expected to possess CI1 type properties [3–6].

Among the CI1 chondrites, the classification for Y-86029 and Y 980115 has been debated because they have both CI- and CM-type features. CY (Yamato-) type was thus newly proposed with 7 more chondrites while it has not been officially admitted yet [4, 7]. The parent bodies of the CY chondrites are considered to shortly experience thermal metamorphism after the aqueous alteration, and the peak metamorphic temperature (PMT) is estimated as 500–900 ℃ [8]. Providing the constraints of PMT is crucial for considering the chemical evolution on the parent bodies, and hence the PMT has been extensively studied for a variety of carbonaceous chondrites [8–14]. For Y 980115, the PMT has been estimated as 500–600 ℃ or > 500 ℃ for a short time (< 100 min) [15], 500–750 ℃ [16], and 700–800 ℃ [17]. These PMTs were usually derived from thermal analysis, such as thermogravimetric differential thermal analysis (TG–DTA) and petrography of the meteorite. However, while the thermal analyses directly provide the higher side constraints of PMT, it has no spatial resolution so that the results sometimes get complicated because of coexistence of various minerals. On the other hand, the petrography is derived from the results of mineral compositions obtained by electron probe microanalyzer (EPMA), backscattered electron (BSE) imaging obtained with scanning electron microscopy (SEM), or SEM–energy-dispersive spectroscopy (EDS), which provide the information of elemental compositions at the probing point. While they are advantageous in terms of their high spatial resolution and high quantitativity, the assignment of the elemental composition to a specific mineral sometimes requires additional information. For example, King et al. found Mg-bearing carbonates on Y 980115, which were assigned as “probably” dolomite since calcite CaCO_3_ and dolomite CaMg(CO_3_)_2_ are the major candidates for the carbonates found on CM and CI meteorites [4]. Thus, some uncertainty may sometimes remain in the mineral identification based on EPMA and BSE. Further, these analytical techniques are destructive, inhibiting the analyses of the whole stone of the meteorite collected. Because the degree of thermal dehydration of Y 980115 is known to be position-dependent [15], it is essential to examine the PMT with non-destructive manner.

As a non-invasive mineral identifier, Raman spectroscopy is a powerful technique. It is readily combined with microscopy, enabling to probe the minerals with (sub-)micrometer spatial resolution. It requires no pretreatment such as surface polishing unlike EPMA or SEM so that it is in principle possible to analyze the whole surface of the sample without damaging it. One of the concerns in this aspect is that too strong laser incidence and/or the laser irradiation in the presence of oxygen sometimes alter the samples [18] so that preliminary experiment is necessary for analyzing rare samples. It would be also noted as the disadvantage of the Raman spectroscopy that its spatial resolution is worse than electron-based techniques such as EPMA, and the vibrational spectroscopy is usually not very suitable to quantifying the elemental composition. Instead, Raman spectroscopy enables to uniquely identify the kind of mineral based on characteristic vibrational bands of each mineral. For example, Raman spectroscopy can identify polymorph of serpentine, which is one of abundant phyllosilicates in matrix of stony meteorites [19, 20]. Further, in some cases, elemental composition of the mineral can be evaluated by wavenumber shift of the characteristic bands. For carbonates, their cation composition is known to be semi-quantitatively obtained from slight shift of the Raman band [21–24]. The quantification of the cation composition based on the Raman spectroscopy is superior to that based on EPMA or SEM–EDS in terms of avoiding so-called matrix effect. Namely, the conventional techniques have sometimes difficulty in distinguish the signal from the aimed mineral and its surrounding matrix if both of them are inside the probing spot. Such matrix effect is totally avoided in the Raman-based quantification because any matrices do not contribute to shift the vibrational frequency of the carbonates.

As for a PMT indicator, carbonates are often used because they are decomposed at several hundred degrees depending on the type of carbonates, and hence the non-invasive and matrix effect-free analysis of carbonates on carbonaceous chondrites are valuable. The matrix effect-free analyses of the cation composition were indeed applied to the carbonates on the Ryugu sand collected by JAXA Hayabusa2 [25]. Considering these analytical advantages, in the present study, carbonates on Y 980115 were analyzed by Raman spectroscopy for estimating its PMT. In results, we found unexpected abundance of nesquehonite that is a kind of carbonates but terrestrial weathering products [26]. Since the carbonates, such as dolomite and calcite, are important minerals for elucidating the thermodynamic conditions where they formed and classifying meteorites, the present research shows the caution on the chemical and thermodynamics analyses of carbonates on primitive meteorites.

## Experimental

Four pieces of Y 980115 collected and stored by the National Institute of Polar Research (NIPR) were used as received. Three of them are smaller than 1 mm and the other is about 5–6 mm. They were analyzed only by Raman spectroscopy. Other than carbonates, a variety of typical minerals on carbonaceous chondrites, such as olivine and magnetite, were found on these chips. Terrestrial dolomites mined at Azcarate Quarry, Navarre, Spain and Butler County, Missouri, USA were analyzed by Raman spectroscopy and TG–DTA as a reference. Note that the Raman spectra of these dolomites are essentially identical [21]. Terrestrial calcite mined in Mexico was also used as a reference. Further, disordered dolomite, in which Ca and Mg atoms are randomly located whereas they are alternately placed layer-by-layer in ordinary dolomite, was synthesized according to literature [27]. Briefly, 0.1 M of MgSO_4_, CaCl_2_, and Na_2_CO_3_ solutions were mixed with a ratio of 1: 1: 2 and stirred for 30 min at 70 °C. After cooling it to room temperature by waiting for 2 h, the precipitate was collected by filtration. The disordered dolomite was analyzed by both the Raman spectroscopy and TG–DTA.

The Raman spectra were observed with a lab-built Raman spectrometer [18] and a commercial apparatus (Raman-11i), both of which were combined with a microscope. The two apparatuses were used because the quality of microscopic images of the lab-built system was much superior to those of the commercial one but its usage requires expertise. The lab-built system was thus used to find out the carbonate grains on the meteorites. While the reference Raman spectra for terrestrial dolomite and calcite were also measured by the lab-built spectrometer for fair comparison, those of dolomites heated by TG–DTA were measured with the commercial system. The wavelength of the excitation laser was 532 nm. The excitation power was 2.2 and 5.0 mW at the sample faces of the meteorites and the terrestrial dolomites, respectively. The incident laser was focused onto the samples with an objective lens (Olympus LUCPlanFLN40x (N.A. 0.60) or Nikon Plan fluor 40x (NA 0.75), for the lab-built or the commercial spectrometers, respectively). The wavenumber of the spectra was calibrated with those of intense Raman bands from a silicon wafer (520.6 cm^−1^) [28] and/or a standard polystyrene plate provided by The National Institute of Advanced Industrial Science and Technology, Japan. No post-processes, such as baseline correction and smoothing, were applied to the spectra except the wavenumber calibration. More than 60 carbonate grains were found and probed on Y 980115.

TG–DTA was performed for about 25 mg of the terrestrial and synthesized (disordered) dolomites with a commercial analyzer (Rigaku; Thermo Plus TG 8120). The terrestrial dolomite was ground with a pestle and a mortar before the TG–DTA measurement. The temperature was raised up to 800 °C with a rate of 10 °C / min.

## Results and discussion

**Fig. 1:**
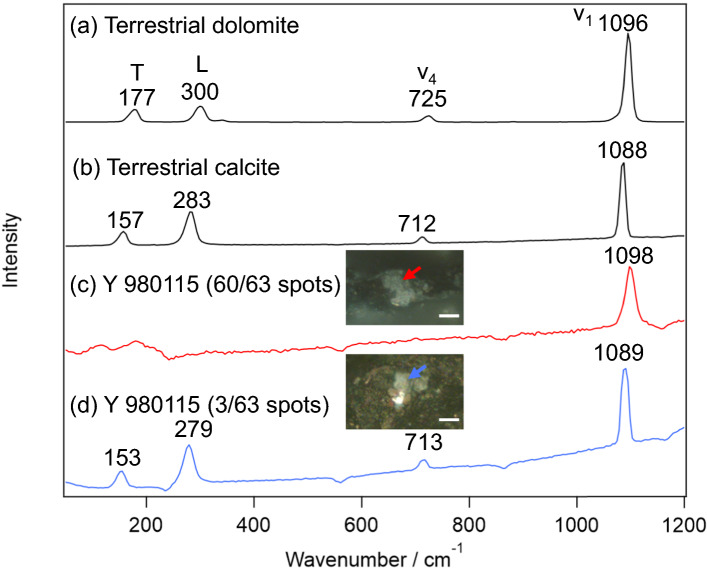
Typical Raman spectra of carbonates. **a**, **b** Terrestrial dolomite and calcite. **c**, **d** Major and minor carbonate species on Y 980115. The microscopic images for the carbonates on which the Raman spectra were measured are also shown. The scale bars in the pictures represent 10 μm

Typical Raman spectra obtained from Y 980115 were summarized in Fig. 1 together with those of the terrestrial dolomite and calcite. As shown in Fig. 1a, b, it is known that the carbonates show four sharp peaks in this wavenumber region; translational (T), librational (L), CO_3_^2−^ bending (ν_4_), and CO_3_^2−^ stretching (ν_1_) modes [29]. The peak wavenumbers of these bands slightly shift depending on the counter cation of the carbonate. As the peak wavenumbers depend on the cation composition, the composition can be estimated from the peak wavenumbers especially of T and L modes with an accuracy of 2 atom% [22]. However, unexpectedly, most of the carbonates (60/63 grains) on Y 980115 (Fig. 1c) only exhibited ν_1_ band although its high wavenumber (~ 1098 cm^−1^) implies that this carbonate is dolomite. Note that the lack of the T and L bands was characteristic for these dolomite-like carbonates, being contrast to that the lattice modes were observed in calcite as usual (3/63 grains; Fig. 1d). While the appearance of ν_1_ band indicates the probed minerals were indeed carbonates, the lack of the Raman bands of lattice modes suggests that their crystal structure is far from ordinary carbonates. Indeed, (1) the ν_1_ band with slightly higher wavenumber than that of dolomite and (2) the lack of T and L bands are the characteristics of nesquehonite (MgCO_3_·3H_2_O or Mg(HCO_3_)(OH)·2H_2_O), whose Raman spectrum was reported in several literature [30, 31]. On the earth, nesquehonite is mainly produced by weathering of ultramafic rocks, which contains olivine (Mg, Fe)_2_SiO_4_ and pyroxene M_2_Si_2_O_6_ (M is Fe, Mg, Ca, and so on) [31]. Because CO_2_ fixation to nesquehonite occurs under atmospheric pressure condition only by flowing CO_2_ gas into Mg-containing solution in short reaction time (< 10 min) [32], its usage as CO_2_ storage is investigated [31]. From the cosmochemical aspect, it is important to note that nesquehonite is often observed on Antarctic meteorites by terrestrial weathering of Mg phyllosilicates [26, 33]. While there has been no report of finding nesquehonite on Y 980115, it is unsurprising that nesquehonite was produced by the terrestrial weathering since the falling year of Y 980115 is not identified. Despite of the consistence to other Antarctic meteorites, it is important to definitely confirm whether the major carbonates found on Y 980115 are indeed not dolomite because the presence/absence of dolomite is an important index to estimate the alteration history.

To confirm whether the major carbonates are nesquehonite or not, the Raman spectra of the carbonate grains on Y 980115 were measured with wider wavenumber region (not shown). If they are nesquehonite, broad and sharp peaks of OH stretching originating in the crystal water should appear at 3244 and 3557 cm^−1^, respectively [31]. However, unfortunately, strong fluorescence background from the matrix inhibited to clearly observe the OH stretching region. Therefore, it is difficult to conclude that they are indeed nesquehonite.

Instead of directly confirming that the carbonates are nesquehonite, it was attempted to prove that they are not dolomite. Namely, lacking the lattice modes (T and L modes) with holding the internal stretching mode (ν_1_) can be also interpreted as the dolomites on Y 980115 are aggregates of amorphous-like nanocrystals, in which the crystal lattice motions no longer exist. Since the parent body of Y 980115 experienced post-hydration thermal alterations presumably owing to minor shock metamorphism, it would be possible for the dolomites to be crashed to nanocrystals/amorphous by the heat and/or the shock pressure. Further, prior to considering such possibility, a possibility that we just failed to find out dolomite grains on Y 980115 must be considered at first. To discard this hypothesis, we collected Raman spectra of carbonate grains on Orgueil, which were also stored by NIPR. As a result, we could easily find many dolomite grains on Orgueil which exhibited typical Raman spectra of dolomite (not shown). It is thus unlikely that we mistakenly failed to observe the dolomite grains only on Y 980115. Note that the “dolomite” grains on Y 980115 found by BSE imaging were as large as tens micrometers [4], indicating they must be found also by optical microscopy. Therefore, the possibility that the dolomites exist as aggregates of nanocrystals is hereafter discussed.

**Fig. 2:**
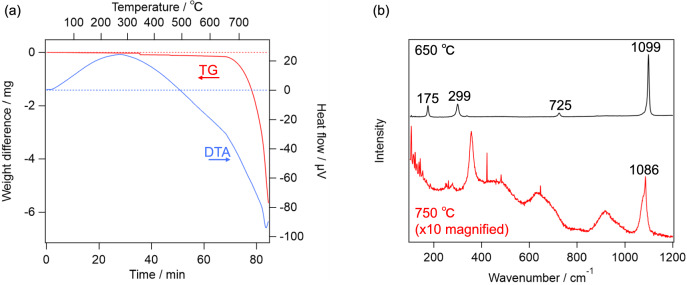
**a** TG–DTA curve of terrestrial dolomite. **b** Raman spectra of the terrestrial dolomite, which were heated up to 650 °C (top) and 750 °C (bottom)

**Fig. 3:**
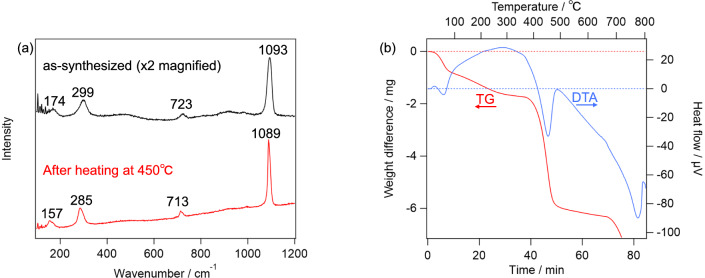
**a** Raman spectra of the synthesized disordered dolomite. Those of the as-synthesized (top) and heated up to 450 °C (bottom) were shown. **b** TG–DTA curve of the disordered dolomite

First, the effect of the heat is considered. As reported in literature [34], dolomite is decomposed into calcite at about 700 °C. As shown in Fig. 2a, TG–DTA of the terrestrial dolomite observed in the present study essentially reproduced those in the literature. If dolomite becomes nanocrystal/amorphous by the heat, Raman spectra of the terrestrial dolomite should become similar to those on Y 980115 after the TG–DTA measurement. However, as shown in Fig. 2b, Raman spectrum of the sample heated up to 650 °C was essentially the same as that of the unheated one, whereas the sample heated up to 750 °C exhibited totally different Raman spectrum from those of ordinary carbonates except calcite-like ν_1_ band at 1085 cm^−1^. These results indicate that the simple heating does not explain the characteristic feature of “dolomite” on Y 980115, whose Raman spectra lacked T and L bands. In addition, we tentatively hypothesized that, dolomite grains produced by the aqueous alteration on Y 980115 was, in fact, disordered dolomite, whose crystal structure was further disrupted by the subsequent post-hydration thermal alteration. Since Ca and Mg atoms in the disordered dolomite are randomly located unlike normal dolomite where Ca and Mg layers are alternately repeated, the disordered dolomite is expected to be more unstable than normal dolomite. Based on this consideration, we synthesized the disordered dolomite, and obtained its TG–DTA curve and Raman spectra. As shown in the top panel of Fig. 3a, the as-synthesized sample provided all of T, L, ν_4_, and ν_1_ peaks in the Raman spectrum. Notably, while the peak wavenumbers of these peaks suggested that the as-synthesized carbonate was indeed dolomite, TG–DTA indicates that the sample was decomposed at around 450 °C as shown in Fig. 3b. Because this temperature is lower than previously estimated PMT of Y 980115, the decomposed product at this temperature is worth being analyzed. Raman spectrum of the heated product at 450 °C is shown in the bottom panel of Fig. 3a, and it clearly suggested that it was calcite. Therefore, while dolomite can be decomposed at the lower temperature than 500 °C if it is disordered, its decomposition cannot explain the Raman spectra obtained from Y 980115. Similarly, the effect of the impact pressure does not explain the lack of the lattice modes of dolomite. According to literature [35], while the Raman intensities for all bands systematically decreased by shock pressure of 68 GPa, band-specific weakening was not observed. By considering these facts, it can be concluded that no dolomite was left on Y 980115, namely, it is suggested that the Mg-bearing carbonates are highly likely to be nesquehonite produced by the terrestrial weathering. One may worry that the nesquehonite found on Y 980115 is extraterrestrial product. While it is difficult to totally exclude this possibility unless isotope measurement is performed, it is unlikely because nesquehonite is known to be formed in only several decades in Antarctica [26].

## Conclusion

In the present study, carbonate grains on Y 980115 were analyzed with Raman spectroscopy because the types of the carbonate left on meteorites are important to estimate the alteration history of the meteorite’s parent body. Unexpectedly, although some literatures found Mg-bearing carbonates by elemental analysis and assigned them to dolomite CaMg(CO_3_)_2_, the Raman spectra of most carbonate grains on Y 980115 only exhibited a band of CO_3_^2−^ symmetric stretching mode and were different from those of typical dolomite. The Raman spectra were compared to those of heated dolomites as well as disordered dolomite, but these reference samples did not reproduce the results on Y 980115. Instead, they were assigned to nesquehonite Mg(HCO_3_)(OH)·2H_2_O which is a typical product of terrestrial weathering for Antarctic meteorites. It is noteworthy that the possibility of the terrestrial weathering on Y 980115 has been hardly discussed while the analyses of Y 980115 have been extensively performed with elemental composition analysis by EPMA or BSE imaging. The present study thus sheds light on the importance of crystallographic information in addition to the elemental composition and TG–DTA analyses.

## Data Availability

The datasets analyzed during the current study are available from the corresponding author on reasonable request.
